# Comment on Lionte et al. Association of Multiple Glycemic Parameters at Hospital Admission with Mortality and Short-Term Outcomes in Acutely Poisoned Patients. *Diagnostics* 2021, *11*, 361

**DOI:** 10.3390/diagnostics11061025

**Published:** 2021-06-03

**Authors:** Vicenç Ferrés-Padró, Silvia Solà-Muñoz, Francesc Xavier Jiménez-Fàbrega, Santiago Nogué-Xarau

**Affiliations:** 1 Advanced Life Support, Emergency Medical Service, Sistema d’Emergències Mèdiques-SEM, 08098 Barcelona, Spain; silviasola@gencat.cat (S.S.-M.); francescxavierjimenez@gencat.cat (F.X.J.-F.); 2 Clinical Toxicology Unit, Emergency Department, Hospital Clínic, 08036 Barcelona, Spain; snoguex@gmail.com

We have read with great interest the article by Lionte et al., “Association of multiple glycemic parameters at hospital admission with mortality and short-term outcomes in acutely poisoned patients”, recently published in your journal [1].

We found it interesting to share similarities and differences. Our team analyzed the performance of prehospital emergency medical service (PEMS) in Catalonia (Spain), with a coverage of 7.7 million inhabitants (18% of the population of the Spanish state), and showed that 80% was performed by basic life support. Poisoning is 3% (5400 cases/year) of the activity involved by the Advanced Life Support (ALS). The mean time of prehospital care was carried out within the first 2.5 h of exposure to the xenobiotic (which ranged from 20 min to 10 h). The main categories of poisons involved were ethanol 38.3%, prescription drugs 28.4%, toxic gases 15%, illicit drugs 11.9% and chemicals (household products, caustics agents or pesticides) 6.4%.

In 2019, ALS internal data exposed poor results of non-invasive determination of blood glucose among those people poisoned or exposed to xenobiotics, including both adult and pediatric patients. Overlooking its systematic determination in the initial care of any critically ill patient represents an easily avoidable risk, due to its accessibility and the ease in interpreting results in any multidisciplinary setting. This shortcoming is especially relevant in the initial care of patients with acute poisoning, due to the limitations of the anamnesis and the need to establish a rapid and reliable differential diagnosis in patients with often complex and multiple clinical symptoms [2,3]. In this regard, the prognostic value of glycemia as a biomarker in some acute, highly lethal levels of poisoning must also be considered [4,5].

For all these reasons, we have recently proposed that the determination of glycemia in poisoned patients should be routine, being included in the panel of indicators of healthcare quality of these patients [6].

In the prehospital setting, it is very difficult to establish what the main cause of the metabolic alteration of glycemia is, mainly because it tends to be multifactorial, both due to a stressful situation and the xenobiotic itself.

Use of a prehospital early warning score (EWS) is controversial due to a lack of evidence of effectiveness in this setting. For instance, there is no evidence on whether patient outcomes differ between a prehospital setting that does and does not use EWS [7]. However, your recent study shows that severe hypoglycemia/hyperglycemia seems to add value in also identifying the risk of short-term morbidity and mortality [8]. All this evidence reinforces your conclusion that the initial blood glucose level is predictive for in-hospital mortality and could provide an early risk assessment tool.

We agree with the authors that the prospective multicenter studies in different geographical areas and by different PEMS and emergency departments are required for the external validation of the utility of all bedside parameters for prehospital adverse events’ use in the poisoned patient.

## Data Availability

Not applicable.
